# Identification of the function and mechanism of m6A reader IGF2BP2 in Alzheimer’s disease

**DOI:** 10.18632/aging.203652

**Published:** 2021-10-27

**Authors:** Yanyao Deng, Hongwei Zhu, Le Xiao, Chao Liu, Ya-Lin Liu, Wenzhe Gao

**Affiliations:** 1 Department of Neurology, The First Hospital of Changsha, Changsha, Hunan Province, China; 2 Department of Hepatopancreatobiliary Surgery, The Third Xiangya Hospital, Central South University, Changsha, Hunan Province, China; 3 Xiangya School of Medicine, Central South University, Changsha, Hunan Province, China

**Keywords:** IGF2BP2, N6-methyladenosine, Alzheimer’s disease, bioinformatics

## Abstract

Alzheimer’s disease, the most common form of dementia in the elderly, is a kind of neurodegenerative disease. However, its pathogenesis and diagnosis remain unclear. M6A is related to nervous system development and neurodegenerative diseases. Here in this study, using multiple RNA-seq datasets of Alzheimer’s brain tissues, along with bioinformatic analysis, we innovatively found that m6A reader protein IGF2BP2 was abnormally highly expressed in Alzheimer’s patients. After compared between Alzheimer’s and normal brain samples, and between IGF2BP2- high and IGF2BP2- low subgroups of Alzheimer’s patients, we took the shared differentially expressed genes as the relevant gene sets of IGF2PB2 affecting Alzheimer’s disease occurrence for subsequent analysis. Then, weight gene correlation analysis was conducted and 17 functional modules were identified. The module that most positively correlated with Alzheimer’s disease and IGF2PB2-high subgroups were mainly participated in ECM receptor interaction, focal adhesion, cytokine-cytokine receptor interaction, and TGF-beta signaling pathway. Afterwards, a hub gene-based model including 20 genes was constructed by LASSO regression and validated by ROC curve for Alzheimer diagnosis. Finally, we preliminarily elucidated that IGF2BP2 could bind with mRNAs in a m6A-dependent manner. This study first elucidates the pathogenic role of IGF2BP2 in Alzheimer’s disease. IGF2BP2 and its relevant m6A modifications are potential to be new diagnostic and therapeutic targets for Alzheimer’s patients.

## INTRODUCTION

About 50 million people worldwide suffer from dementia, and one case of dementia occurs every 3 seconds [1]. Alzheimer’s disease (AD), the most common form of dementia in the elderly, is a kind of neurodegenerative disease. More than 20 million people worldwide are suffering from AD [2, 3]. AD usually manifests symptoms such as cognitive impairment and memory loss [4, 5], and the main pathological manifestation of AD is insoluble neurotoxic aggregates accumulation, including extracellular amyloid plaques formed by amyloid-β and neurofibrillary tangles in nerve cells composed of tau protein [6]. Studies have shown that people with abnormal amyloid have more rapid progression of cognitive decline than that without biomarker evidence of amyloid-β deposition [7–9]. Though amyloid-β can be regarded as the first biomarker becoming abnormal in AD patients [10–13], both amyloid-β and tau protein deposits are required for neuropathologic diagnosis [14, 15]. The traditional diagnosis still has some limitations. So far, the specific pathogenesis of AD is still largely unknown, such as how these proteins are related to each other, and what cause them to accumulate to such destructive levels [1], and its more exactly biological diagnosis is still on the way. Additionally, gene and protein expression profiles changes support the dysfunction during many basic cellular processes in AD pathogenesis [16]. One of the potential mechanisms for altered expression of AD-related genes involves disruption of the epigenome through disease-specific changes in chromatin structure and/or transcriptional programming [17]. These include changes in DNA methylation [18, 19] and histone modification [20–22]. Besides, the diagnosis of AD still faces great difficulties, in which biomarkers are more important than clinical manifestations to provide a breakthrough basis [1].

Chemical modifications in RNA have become an important mechanism for controlling gene expression and protein translation [23]. N6-methyladenosine (m6A) is the most common and reversible post-transcriptional modification of eukaryotic mRNA [24], and it is also a multifunctional regulator of mRNA splicing, localization, translation and stability [25, 26]. Methyltransferases (regarded as m6A “writers”), demethylases (regarded as m6A “erasers”), and binding proteins (regarded as m6A “readers”) selectively recognize methylated RNA to perform regulatory functions [27]. The brain is rich in m6A which plays a broad role in adult brain development and function [28–30]. M6A is proven to be related to nervous system development and neurodegenerative diseases [31–33]. M6A RNA methylation is considered to be a new frontier in neuroscience, which can provide us with a new perspective in the understanding of neurodevelopment and neurological diseases. Moreover, increasing evidence has revealed that the m6A signaling pathway is closely associated with learning and memory, whose impairments are typical clinical manifestations of AD [32, 34–38]. On the other hand, as a neuron surface protein, m6A can promote axon growth, synapse formation and spine induction [39–41]. Insulin-like growth factor 2 mRNA binding protein 2 (IGF2BP2) acts as a reader to regulate m6A [42]. However, whether IGF2BP2 can be used as a marker and auxiliary diagnosis of AD remains to be determined.

Based on the Gene Expression Omnibus database (GEO), we identified m6A regulator IGF2BP2, whose increase is closely related to AD. Furthermore, we conducted modular exploration to explore the potential mechanism of IGF2BP2 in AD. Finally, we constructed and validated a diagnostic model for AD. Our findings suggested that IGF2BP2 may serve as a novel diagnostic biomarker and its increase might associated with AD through m6A mechanism. The gene-based diagnostic model provided us a new way on accurate diagnosis of AD.

## RESULTS

### M6A reader gene IGF2PB2 was highly expressed in AD

**Figure 1:**
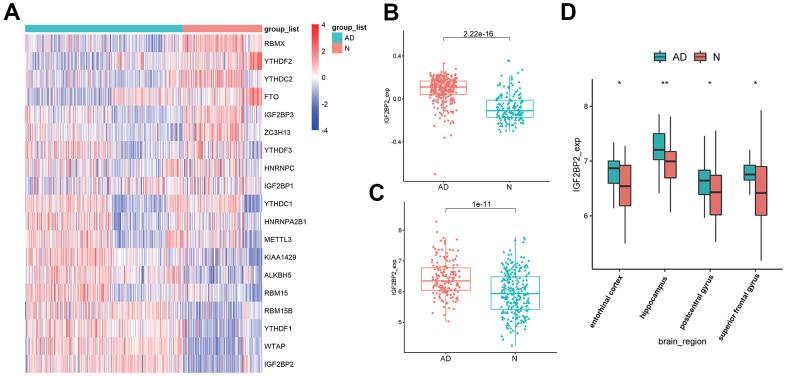
**Identification of DEGS in AD.** (**A**) The expression of 19 m6A modification-related genes in GSE33000. (**B**) IGF2BP2 expression in AD patients and in normal people in GSE33000. (**C**) IGF2BP2 expression in AD patients and in normal people in GSE48350 and GSE5281. (**D**) IGF2BP2 expression in different brain regions of AD patients in GSE33000.

Based on published literature [43, 44], we brought 19 m6A-related genes into our analysis, and the expression levels of these 19 genes were extracted from 624 samples of GSE33000. Differentially expressed analysis showed that among the 19 m6A regulators, the expression of IGF2PB2 in AD patients was significantly higher than that of normal brains (Figure 1A). Further comparison of IGF2PB2 expression between AD and normal samples in three data sets (GSE33000, GSE48350 and GSE5281) also showed that IGF2BP2 expressed more in AD patients than in normal people (GSE33000: log2FoldChange = 0.170, p<2.22e-16, Figure 1B; GSE48350 and GSE5281: log2FoldChange = 1.488, p=1e-11, Figure 1C). Moreover, considering the heterogeneity of brain tissue, we also explored the expression of IGF2BP2 in different brain regions in GSE48350. It showed that IGF2BP2 was highly expressed in entorhinal cortex, hippocampus, postcentral gyrus and superior frontal gyrus in AD patients compared with normal tissue (Figure 1D). These results preliminarily indicated that IGF2PB2 was abnormally highly expressed in AD patients, this phenomenon might be related to the pathogenesis and development of AD.

**Figure 2:**
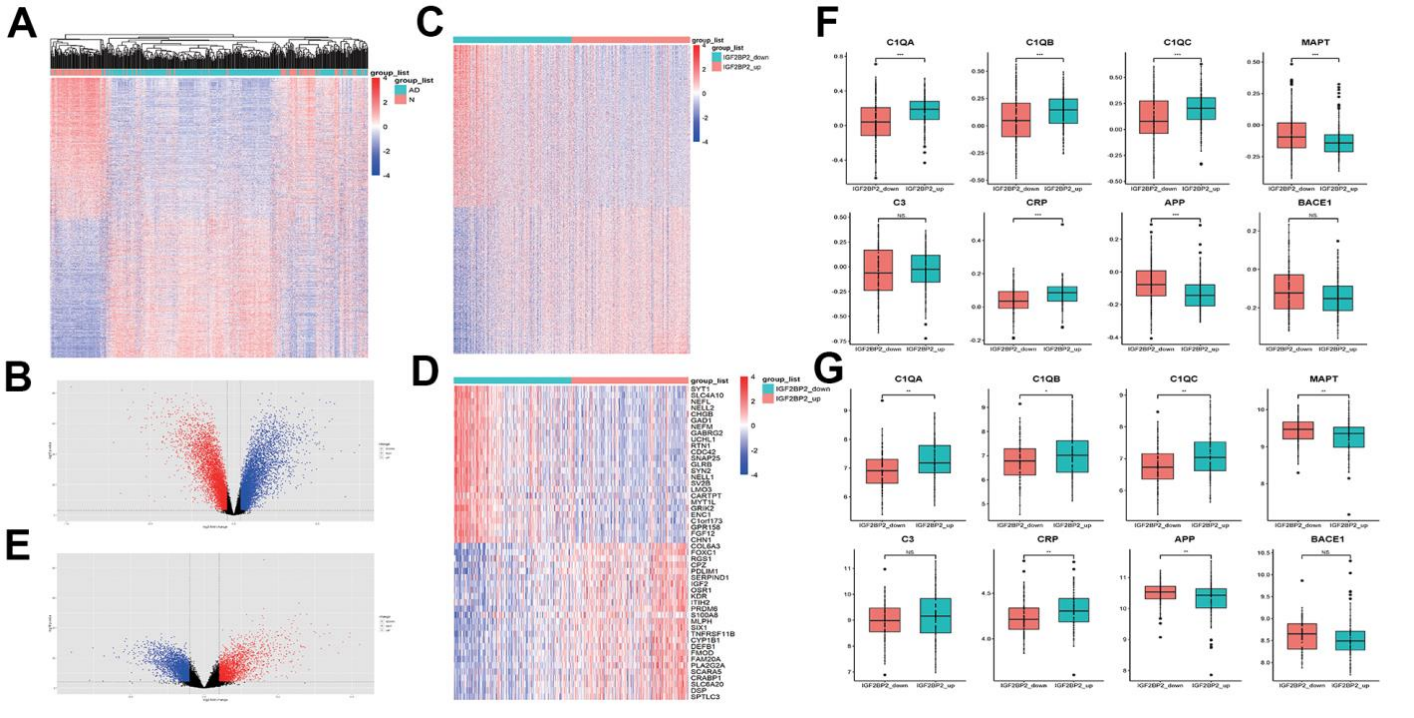
**Identification of DEGS related with IGF2PB2 in AD.** (**A**, **B**) DEGs between AD patients and normal people in GSE33000. (**C**, **E**) DEGs between IGF2PB2 high and low group in AD patients in GSE33000. (**D**) The top25 increased differentially expressed genes and the top25 decreased differentially expressed genes between IGF2PB2 high and low group in AD patients in GSE33000. (**F**, **G**) Expression status of systemic inflammation biomarkers and AD pathological markers in IGF2BP2_high and IGF2BP2_low subgroups of AD patients in GSE33000 (**F**) and GSE48350/5281 (**G**) datasets.

To further find genes that related to IGF2BP2 in AD, first of all, we obtained 13968 differentially expressed genes (DEGs) between AD and normal tissues in GSE33000 (Figure 2A, 2B). Among them, there were 6918 increased genes and 7050 decreased genes in AD patients. Then, we divided AD patients into two groups according to the median expression level of IGF2PB2 and compared to get 9908 DEGs related to IGF2PB2 expression status (Figure 2C–2E). After intersecting the two DEG sets, we took the shared genes as the relevant gene sets of IGF2PB2 affecting AD occurrence for subsequent analysis.

Given that IGF2BP2 had obvious abnormal expression in AD, we also explored the expression of canonical systemic inflammation biomarkers as well as AD pathological markers [45, 46] in IGF2BP2_high and IGF2BP2_low subgroups defined by the median expression value of IGF2BP2 in both GSE33000 and GSE48350/5281 datasets (Figure 2F, 2G). It turned that the expression of Complement component C1Q (C1QA, C1QB and C1QC) and C reactive protein (CRP) was significantly higher in IGF2BP2_high subgroup in AD patients, with no significant difference in C3, suggesting that IGF2BP2 could exacerbate the inflammatory response in the brain tissue of AD patients. Furthermore, the mRNA expression level of β amyloid (Aβ) and Tau protein, showed by their corresponding genes APP and MAPT, was abnormally lower in IGF2BP2_high subgroup, but the expression of BACE1, the key lyase in the formation of Aβ was not influenced. These results indicated that IGF2BP2 high expression might correlated with the increased expression of APP and MAPT gene variants rather than the normal genes, but further results based on next-generation sequencing were needed to verify our hypothesis.

### Module associated with IGF2BP2 in AD

**Figure 3:**
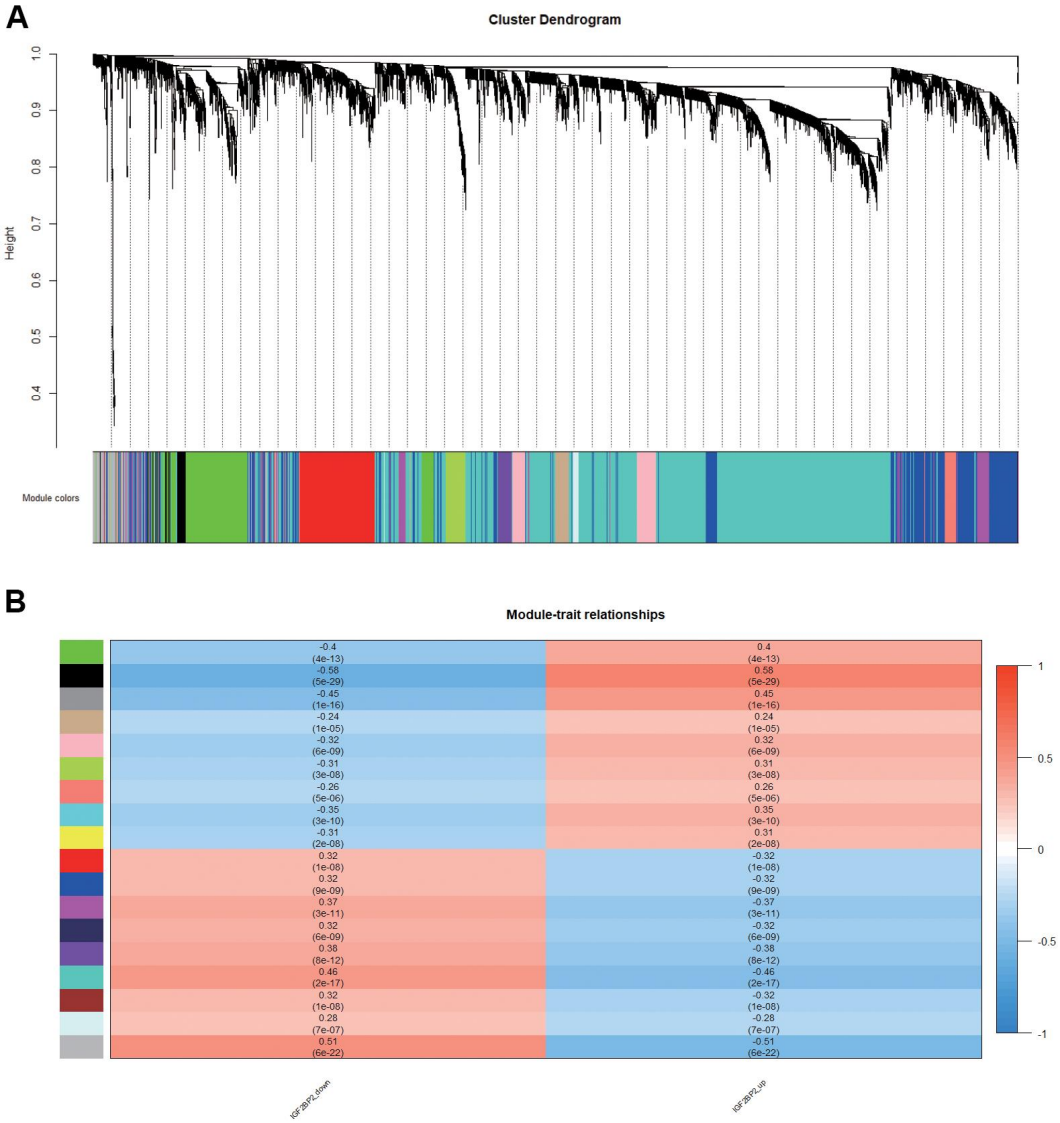
**Module associated with IGF2BP2 in AD.** (**A**, **B**) 17 modules related to the expression level of IGF2PB2 analyzed by WGCNA.

In order to find the key modules most relevant to IGF2BP2 expression status in AD, we performed weight gene correlation analysis (WGCNA) using the expression profiles of the abovementioned DEGs which related to the expression level of IGF2PB2, thereby identifying 17 modules (Figure 3A). Among them, the black module composed of 440 genes had the most significant positive correlation with the increase of IGF2BP2 expression (correlation coefficient=0.58, P=5E-29; Figure 3B), while the turquoise module had the most significant negative correlation (correlation coefficient=-0.46, P=2E-17; Figure 3B). Since IGF2BP2 recognized m6A-modified RNA to enhance its stability and increase translation, we paid more attention to the module that had a positive correlation with IGF2BP2 expression, which was the black module. Finally, according to GS>0.4 and MM>0.9, the 440 genes in the black module were further reduced to 65 genes, which were defined as the hub genes with the strongest positive correlation with IGF2BP2 expression.

### Pathway analysis for hub genes that positively related to IGF2BP2

**Figure 4:**
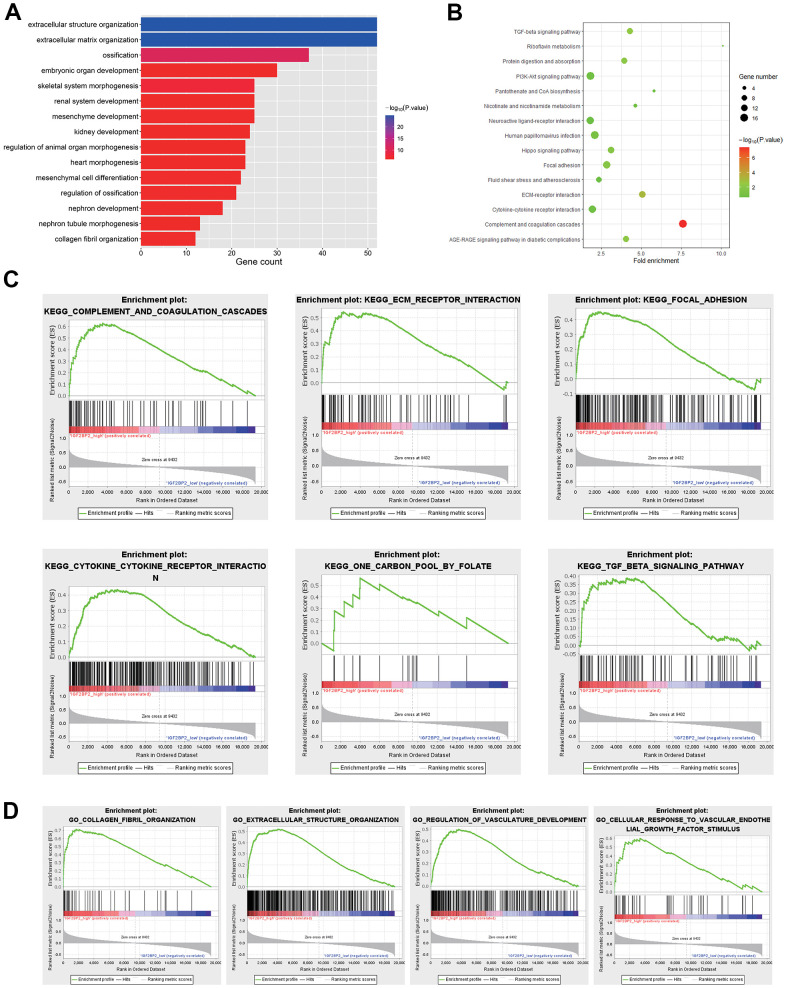
**Pathway analysis in DEGS.** (**A**) Biological processes of 440 DEGs in black module. (**B**) KEGG analysis of 440 DEGs in black module. (**C**) KEGG pathways enriched in IGF2BP2-high by GSEA. (**D**) GO biological processes enriched in IGF2BP2-high by GSEA.

GO and KEGG analysis for 440 genes in the black module were first conducted. Results showed that DEGs was significantly enriched in biological processes such as extracellular structure organization and extracellular matrix organization by GO term enrichment analysis (Figure 4A). In addition, KEGG analysis indicated that DEGs was most significantly enriched in complement and coagulation cascades related pathways (Figure 4B). To further explore the influence of IGF2BP2 expression status on the enrichment of signaling pathway, the AD patients in GSE33000 were divided into IGF2BP2 high and low groups, with the whole transcriptome subjected to GSEA. The results indicated that pathways including complement and coagulation cascades, ECM receptor interaction, focal adhesion, cytokine- cytokine receptor interaction, one carbon pool by folate, TGF-beta signaling pathway were significantly enriched in IGF2BP2_high subgroup (Figure 4C). On the other hand, as for GO biological process enrichment, GSEA results were enriched in collagen fibril organization, extracellular structure organization, regulation of vasculature development, and cellular response to vascular endothelial growth factor stimulus (Figure 4D). The common enrichment pathways in the above results, such as ECM receptor interaction, focal adhesion, cytokine-cytokine receptor interaction, and TGF-beta signaling pathway, may be closely related to the effect of IGF2BP2 on AD, thus providing us some insight into the biological effects associated with IGF2BP2.

### Construction and validation of the IGF2BP2-related AD diagnosis model

**Figure 5:**
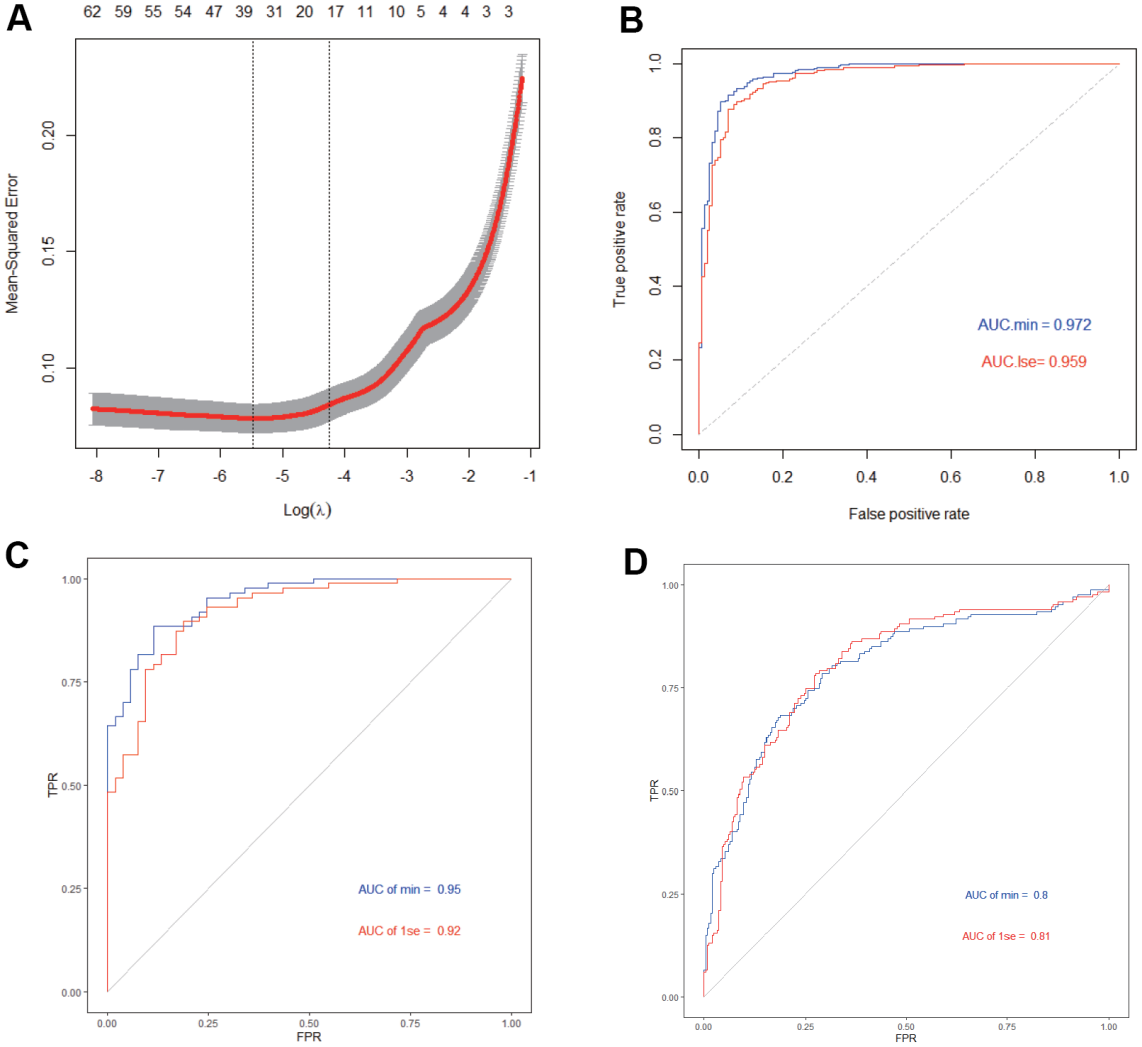
**Construction and validation of AD diagnosis model.** (**A**) LASSO model. (**B**) ROC curves analysis in train set. (**C**) ROC curves analysis in test set. (**D**) ROC curves analysis in validation.

We then performed lasso regression on IGF2BP2 and the 65 hub genes in black module to construct a diagnostic model for AD. Based on the λ value of LASSO regression, two diagnostic models have been constructed: the first one is the model constructed with 38 genes that maximized the predictive performance regardless of the number of genes, and the second one is the model obtained by including a minimum of 20 of the most critical genes at the appropriate sacrifice of diagnostic efficiency (Figure 5A). In addition, we randomly sample 70% of the samples in GSE33000 as the train Set, and the remaining 30% as the test Set to judge the internal diagnostic stability of this model. We found that in the train set the AUC of the first model was 0.972, while the second model was 0.959, which was still a relative high value (Figure 5B). On the other hand, in the Test set the AUC of the first model was 0.95, and the second model was 0.92 (Figure 5C). Moreover, we used the data in GSE48350 and GSE5281 as the external validation set, with the AUC of the first model 0.80, the second model 0.81 (Figure 5D). In summary, the 20-gene-model could be used to construct a suitable AD diagnosis model with the least hub genes while maintaining relatively high diagnostic efficiency. This model included the following genes: TNFRSF11B, GYPC, TP53I11, F10, PKP2, FCGRT, PRRX2, PRELP, BMP6, MRC2, AOX1, PRDM6, STRA6, PTGFR, CFH, SLC22A3, LOC388630, ANXA2P2, COL1A1 and IGF2BP2.

### M6A-dependent mechanism between IGF2BP2 and genes in AD diagnostic model

**Figure 6:**
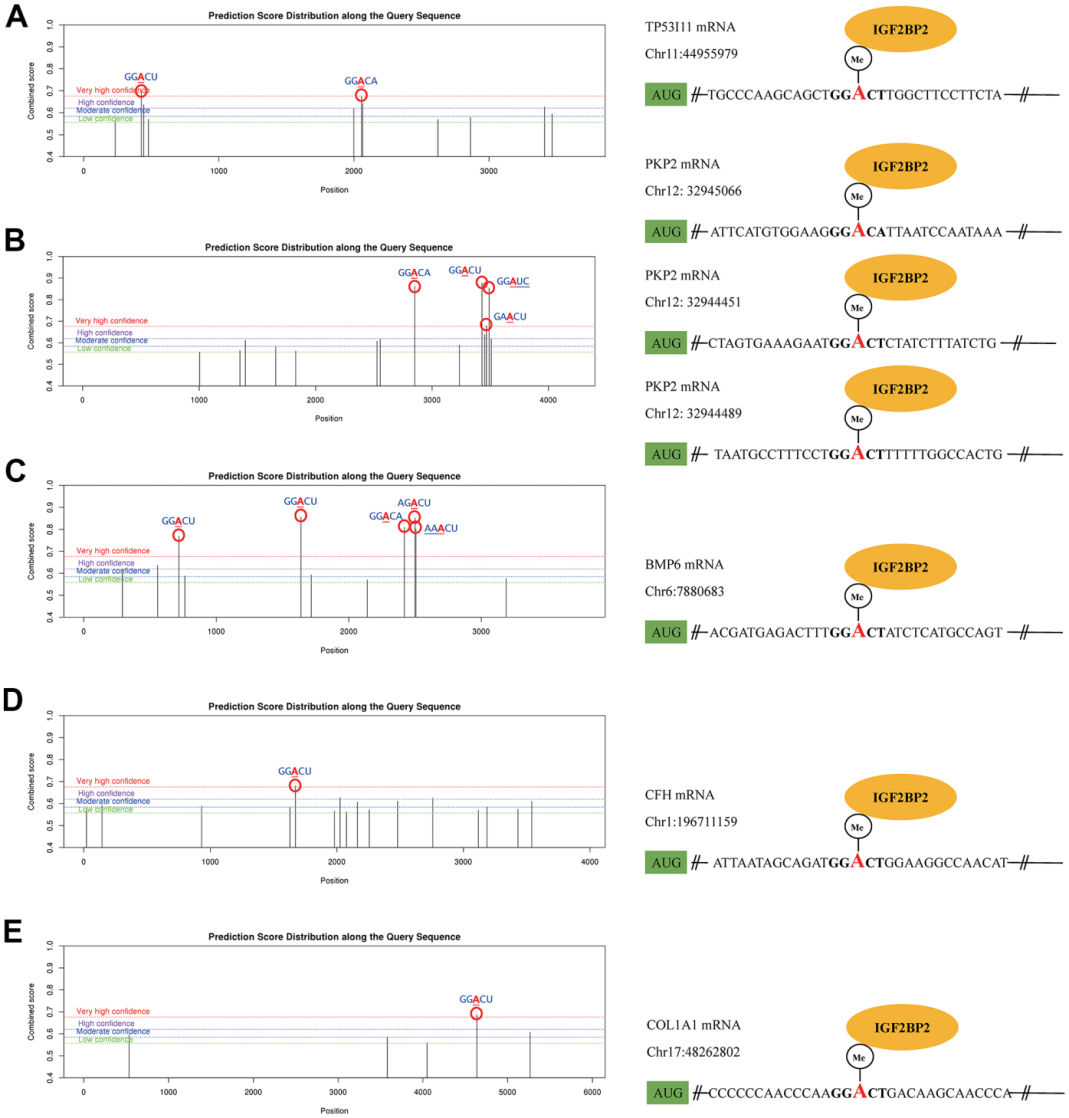
**The m6A mechanism of IGF2PB2 and its downstream mRNA targets.** The m6A mechanism of IGF2PB2 and TP53 (**A**), PKP2 (**B**), BMP6 (**C**), CFH (**D**), COL1A1 (**E**).

As a classical RNA binding protein, IGF2BP2 was identified as a m6A reader protein in 2018. The binding between IGF2BP2 and its targets could increase the stability of mRNAs, thereupon promote the expression of the downstream mRNAs and proteins. After WGCNA and LASSO analysis, we got an IGF2BP2-related diagnostic model containing 20 genes. Here we presumed that IGF2BP2 might specifically bind with some of these genes in a m6A-dependent manner. To explore this mechanism, we first searched the possible genes that contained m6A modification sites with high confidence in 20 hub genes using SRAMP. Then, in RMBase, we further predicted the specific binding of IGF2BP2 to m6A sites of target mRNAs. Ultimately, TP53I11, PKP2, BMP6, CFH and COL1A1 were preliminarily confirmed to possess “very high confidence” m6A binding sites with IGF2BP2 (Figure 6A–6E). These binding sites had the value of subsequent experimental verification, and might be able to explain the molecular mechanism of IGF2BP2 promoting the pathogenesis and development of AD.

## DISCUSSION

Accumulation studies have shown that m6A, as a dynamic and reversible regulatory biomarker [24], is related to nervous system development and neurodegenerative diseases [31–33], but its regulatory mechanism is still unclear. Experiments have confirmed that m6A epitranscriptome plays an important part in synaptic plasticity, neuron development, stress response and cognition [28, 29, 47, 48]. M6A signaling influences learning and memory. Given that learning and memory disorders are clinical features of AD, decrease of m6A signaling might be related to the pathophysiology of AD [49]. M6A methylations may be potential biomarkers for cognitive dysfunction such as AD, mild cognitive impairment, and vascular dementia [50]. Mutations in genes which encoding the neuronal glycoprotein m6A have link with psychiatric disorders such as Alzheimer’s disease [51]. In previous studies, pathway analyses have predicted the potential effect of m6A methylation RNA in AD, and the high-throughput sequencing of m6A RNA methylation alterations in AD and C57BL/6 mice was also verified this association [52, 53]. Besides, m6A controls key gene expression in AD- related pathways, which indicates that m6A renders its essential roles in aging and neurodegenerative diseases [54]. In our study, we found that IGF2BP2, as a m6A reader, increased in AD patients than in normal people, which might indicate a potential relationship with the occurrence of AD.

As a reader of m6A, IGF2BP2 is associated with carcinogenesis, and has been tested to be associated with the occurrence and prognosis of various cancers including colorectal cancer [55], gastrointestinal cancer [56], endometrial cancer [57], pancreas cancer [58], breast cancer [59] and Head and Neck Squamous Cell Carcinoma [60]. During the progression of colorectal cancer, LINRIS prevents K139 ubiquitination of IGF2BP2, thereby maintaining its stability. This process prevents IGF2BP2 degradation through the autophagy-lysosomal pathway, which is further related to MYC-mediated glycolysis. Besides, studies confirmed that miR-34a silence resulted in IGF2BP3 activation, which was related with gastric tumorigenesis and differences in prognosis [61]. However, its association with AD is not clear. We further explored the genes associated with differential expression of IGF2BP2, and a total of 17 AD-related modules were identified based on WGCNA data. Furthermore, according to GS>0.4 and MM>0.9, a total of 65 hub genes were identified eventually.

We further explored the potential pathways related to AD. Pathway enrichment analysis indicated that gene modules related to IGF2BP2 are significantly enriched in ECM receptor interaction, focal adhesion, cytokine-cytokine receptor interaction and TGF-beta signaling pathway, which may participate in the occurrence of AD. Many pathways have been confirmed to be related to AD. For example, increasing studies have found that the expression of cytokine-cytokine receptor interaction pathway in AD is increased [62, 63]. Moreover, TGF-beta signaling pathway may serve as a neurotrophic pathway that satisfies the important protective and survival effects of neurons. It acts as a potential target of neurodegenerative diseases, and is critical in the pathogenesis of AD [64, 65].

In addition, we used IGF2BP2 and 65 hub genes to construct a 20-gene-based diagnostic model. Both training set and test set showed a high AUC value with this lasso model using ROC curve analysis, which is also verified in an independent verification dataset. This meant our diagnostic model might provide a novel idea for the diagnosis of AD.

Finally, we searched for the possible m6A mechanism of IGF2PB2 in 20 hub genes. Further studies have shown that TP53I11, PKP2, BMP6, CFH and COL1A1 had m6A and could specifically bind with IGF2PB2. It can be seen that IGF2PB2 probably acts as m6A “reader” that binds to TP53I11, PKP2, BMP6, CFH and COL1A1 m6A, and ultimately leads to AD. Nevertheless, we did not find that the remaining hub genes had m6A or specific binding to IGF2PB2, which might be explained by other function of IGF2PB2 [66, 67].

Our research used bioinformatics methods to reveal the possible molecular mechanisms and built a diagnostic model for AD. However, more key genes and specific signaling pathways underlying AD need to be further verified through cell and animal experiments. On the other hand, to what extent the increase of IGF2BP2 expression in AD patients promotes the occurrence and development of AD still remains to be tested.

In summary, our study proved that high expression of IGF2BP2 may associate with the increase of the PKP2 through m6A association mechanism, which may further lead to the occurrence of AD. Besides, a diagnostic model was constructed and validated based on that.

## MATERIALS AND METHODS

### Datasets acquisition

Three data sets (GSE33000, GSE5281 and GSE48350) were selected from GEO database (https://www.ncbi.nlm.nih.gov/geo/) for this study. Among them, GSE33000 was based on GPL4372 platform including postmortem prefrontal cortex samples of 310 AD patients and 157 normal people. The “getGEO” function from “GEOquery” package in R was applied for data downloading of this dataset. Besides, GSE5281 included brain tissue samples of 87 AD patients and 74 normal people, GSE48350 included 80 AD patients and 173 normal people. Both datasets were based on GPL570 platform. Thus, the “getGEOSuppFiles” function from “GEOquery” package was applied for downloading the raw data of these datasets, and the “justRMA” function from “affy” package was applied for the normalization of them.

### Differentially expressed genes (DEGs) identification

The “limma” package from R was applied for DEG analysis in this study. For the selected datasets, we select the median expression of IGF2BP2 as the cutoff point to divide AD samples into IGF2BP2_high and IGF2BP2_low subgroups. DEG analysis was then conducted between AD and normal brain tissues and between IGF2BP2_high and IGF2BP2_low subgroups by lmFit and eBayes methods. P < 0.05 adjusted by the false discovery rate (FDR) was considered as significant. The intersection of the DEGs obtained from the two analyses was defined as IGF2BP2-related AD genes.

### Weight gene correlation network analysis (WGCNA) and hub genes identification

After getting IGF2BP2-related AD genes, WGCNA analysis was performed using “WGCNA” package in R for GSE33000 dataset. Briefly, “hclust” function was used to hierarchical clustering analysis; then, the soft thresholding power value was calculated by “pickSoftThreshold” function; next, the “blockwiseModules” function was used for constructing co-expression network in IGF2BP2_high and IGF2BP2_low subgroups of AD patients, the module that was mostly correlated with IGF2BP2_high or IGF2BP2_low subgroups was defined as the most valuable module and was selected for further screening. Gene Significance (GS) > 0.4 and Module Membership (MM) > 0.9 was considered as threshold to screen hub genes in the most valuable module.

### Gene Ontology (GO), Kyoto Encyclopedia of Genes and Genomes Pathway (KEGG pathway) and Gene Set Enrichment Analysis (GSEA)

Using enrichGO and enrichKEGG function in “clusterProfiler” package, GO and KEGG pathway analysis were performed based on genes in the most valuable module in WGCNA analysis. Furthermore, Gene Set Enrichment Analysis (GSEA) provided by the JAVA program (Version 4.0.3) with MSigDB v6.1 was applied to explore the downstream biological processes affected by differential expression of IGF2BP2.

### Construction and validation of the diagnostic model for AD

To build a diagnostic model that could maximally distinguish AD from normal brain samples, least absolute shrinkage and selection operator (LASSO) regression was applied using “glmnet” package to select most representative features in hub genes obtained from the most valuable module in WGCNA analysis. A model index for each sample was created using the regression coefficients from the LASSO analysis to weight the expression value of the selected genes with the following formula:


$$Diagnostic\;Index=\sum_{i=1}^{n}Coef_{i}*x_{i}$$

where Coef_i_ means the coefficients for each gene, x_i_ is the expression value of each gene.

Then, samples in GES33000 dataset were randomly assigned to the training set (70%) and test set (30%) for internal test. GSE5281 and GSE48350 datasets were applied for external validation of our diagnostic model. The “ROCR” package was used for drawing ROC curves for the training, testing and validation datasets respectively.

### The m6A mechanism between IGF2PB2 and hub genes

To initially explore whether IGF2BP2 and hub genes from WGCNA and LASSO analysis could constitute a m6A-dependent mechanism, SRAMP online database (http://www.cuilab.cn/sramp) and RMBase V2.0 online tools were applied. SRAMP could predict the precise location of m6A modification on mRNAs and provide the confidence of every modification site, while RMBase V2.0 could further inquire whether these modification sites could be specifically recognized and bounded by IGF2BP2.
